# A Survey of Dentists in the Management of Dentine Hypersensitivity: A Questionnaire-based Study

**DOI:** 10.1055/s-0039-1694306

**Published:** 2019-09-19

**Authors:** Chrysanthie Exarchou, Ioanna Betsani, Dimitra Sakellari, Dominiki Chatzopoulou, David Gillam

**Affiliations:** 1 Department of Preventive Dentistry, Periodontology and Implant Biology, School of Dentistry, Aristotle University of Thessaloniki, Thessaloniki, Greece; 2 Institute of Dentistry, Barts and the London School of Medicine and Dentistry, Queen Mary University London, London, England

**Keywords:** etiological factors, dentine hypersensitivity, management, quality of life, questionnaire

## Abstract

**Objective** Previous studies have indicated that dentists may be uncertain about the etiology, diagnosis, and effective management of dentine sensitivity/dentine hypersensitivity (DH) **.** The purpose of the present study was to evaluate the knowledge and understanding of Greece-based dental professionals in treating DH **.**

**Materials and Methods** A 26-item questionnaire was sent to a representative sample of Greek dentists.

**Results** Two hundred thirty questionnaires were originally provided to the participants and of the 210 questionnaires that were returned, 191 questionnaires (90 M; 86 F; mean age 36.26 years [standard deviation: 11.34]) were included for analysis, a response rate of 83% was observed. 39.8% of dentists indicated that 1 in 10 of their patients experienced discomfort from DH with 76.4% of dentists indicating that their patients initiated the conversation on DH. In contrast, 44% of the dentists indicated that they initiated the relevant conversation. 34.9% of dentists indicated that the duration of discomfort lasted up to 3 weeks and 76.4% indicated that DH had an impact on their patients’ quality of life. Incorrect tooth brushing was considered to be a major etiological factor (68.6%) with “air blast” (37.3%) and “probing” (15%) as the main methods for identification. 83.6% of dentists indicated that they were confident in recommending over-the-counter products for home use.

**Conclusion** The results of this study suggest that in terms of knowledge and understanding of DH, there is still confusion concerning some aspects of the diagnosis and management of the condition.

## Introduction

According to the consensus-based recommendations by the Canadian Advisory Board on Dentin Hypersensitivity, dentine hypersensitivity (DH) may be defined as “a distinctive short sharp pain arising from exposed dentine, characteristically in response to an array of stimuli including thermal, tactile, evaporative, osmotic or chemical, which cannot be attributed to any other form of dental defect, disease or pathology.” 1 It is also evident from the published prevalence studies that DH can affect the quality of life (QoL) of those who suffer discomfort from the condition. 2 3 4 There are, however, discrepancies in the reported prevalence of DH that can range from 1.1 to 98% depending on the methodology employed (questionnaire vs. clinical examination) as well as the location (university clinic, dental practice, consumer groups) and the cultural environment. 5 However, an overview on the burden of DH by Cunha-Cruz and Wataha appeared to indicate that the prevalence was in the region of 10% of the general population. 6 There are several outstanding issues that need to be resolved when evaluating the management of DH 7 , for example, 1 is the condition under- or overestimated by dentists, 2 is the condition adequately diagnosed and successfully managed by dentists in daily practice, 3 is the impact of DHS on the QoL of sufferers adequately diagnosed and treated, and 4 is the condition adequately monitored by clinicians in daily practice. These concerns have been addressed to some extent in previous studies, although it is clear that clinicians are still uncertain about the etiology, diagnosis, and effective management of DH. 5 7 8 9 10 11 12 13

The purpose of the present questionnaire-based study was to evaluate the knowledge and understanding of Greece-based dental professionals in treating DH.

## Materials and Methods

A 26-item questionnaire on DH that included both multiple choice and open-ended questions was handed to a representative sample of Greek dentists either general dental practice or undertaking postgraduate studies. The questionnaires were handed out within the Dental School as well as a National Dental Conference. The study was approved by the Ethics Board/Hospital Committee at the School of Dentistry, Aristotle University of Thessaloniki, Thessaloniki, Greece (# 42,26/6/2017).

The questionnaire was based on a previously validated questionnaire used in the United Kingdom related to the understanding of DH 8 14 15 and has been recently updated and used in several studies namely in Brazil, India, and Kuwait. 5 16 17

The questionnaire was designed based on worldwide reports about DH including its prevalence, the important predisposing factors, major triggers, mechanisms, differential diagnosis, patient management, dentist management, and continuing education related to DH.

The first section of the questionnaire was designed to check the demographic characteristics of dentists and included years in practice, number of DH cases dentists treated in their practices, their initial approach to such cases, and the impact of DH on the QoL of their patients. The final section focused mainly on the dentists’ perspective of their patients presenting with DH and its causes, triggers, and predisposing factors as well as diagnosis and management. The original English questionnaire was translated into Greek and then back into English to ensure that the meaning remained the same.

### Inclusion Criteria

All dental professionals practicing in Thessaloniki, Greece, were willing to participate in the study.

## Results

Two hundred thirty questionnaires were originally provided to the participants and of the 210 questionnaires that were returned, 191 questionnaires (90 M; 86 F; mean age 36.26 years [standard deviation, SD: 11.34]) were included for analysis, a response rate of 83%.

Data were collected over a period of 6 months from June 30, 2017, to December 30, 2017. Data were entered and the results were analyzed using SPSS 22.0 for Windows (IBM, Portsmouth, United Kingdom) in the form of frequency distribution tables and presented as pie charts.

The mean years from graduation were 13.44 years (SD: 11.36) (70 missing values). One-hundred thirty-two (72.9%) of the participants were general dentists and 49 (27.1%) participants were postgraduate students (10 missing values). One hundred five (54.9%) participants reported that they had a dental specialty that included mainly prosthodontics ( *n* = 19; 18.1%], oral surgery ( *n* = 11; 10.5%), and periodontics ( *n* = 10; 9.5%).

In response to question 2 (Q.2) on whether they had seen one or more patients reporting DH in the weeks prior to the appointment (2–4 weeks/month), 76.4% ( *n* = 146) say they had seen a patient with DH during this period; 18.8% ( *n* = 36) did not see any patients with 4.7% of dentists not sure or could not remember whether they had seen any patients with DH during this time.

When asked to respond to Q.3 on the percentage of dentate patients who experienced DH, 39.8% ( *n* = 76) of dentists indicated that one in 10 of their patients experienced discomfort from DH with 25.7% ( *n* = 49) indicating that one in 20 of their patients experienced discomfort from DH.

Dentists were then asked to indicate whether the patient initiated the conversation on whether they were experiencing discomfort from DH (Q.4). About 76.4% ( *n* = 146) of dentists indicated that their patients initiated the conversation with 15.2% ( *n* = 29) indicating that the patient did not initiate the conversation with 8.4% ( *n* = 16) unable to remember. When asked to state whether they initiated the conversation, 44% ( *n* = 84) said they initiated the conversation with their patients, 11.2% ( *n* = 22) did not; 38.2% ( *n* = 73) of dentists responded that they sometimes initiated the conversation about DH with 6.3% ( *n* = 12) of the dentists not responding.

When asked whether they routinely observed signs of DH during their examination of their patients (Q.6a), 60.2% ( *n* = 115) stated they did look for signs of DH during the examination; 14.1% ( *n* = 27) did not; 21.5% ( *n* = 41) responded that they sometimes looked for signs with 3.6% ( *n* = 7) not sure or did not know whether they looked for signs of DH with one missing value (0.5%). The responses for Q.6b indicated that most dentists (85.9% [ *n* = 164]) used an air blast to detect DH with 33.5% ( *n* = 64) using a dental probe. Other responses included using a water spray from the dental syringe (14.7%; *n* = 28); a combination of methods (5.2%, *n* = 10), ice stick (12.6%; *n* = 24), a pulp tester or a dental scaler (2.6%; *n* = 5), and a nonspecified method (0.5%; *n* = 1).

**Fig. 1:**
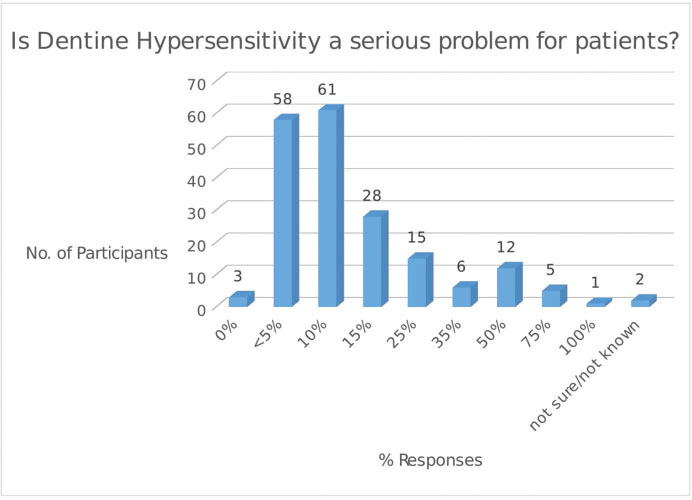
Dentists’ response to Q.7: Is dentine hypersensitivity a serious problem for patients?

The response to Q.7 regarding the seriousness of DH in their patients indicated that 86.4% ( *n* = 165) of dentists considered DH to be a serious problem for 25% of their patients, although 63.9% ( *n* = 122) indicated that it was only a serious problem for 1 in 10 of their patients ( Fig. 1 ).

When asked about how long patients suffered with DH (Q.8), 34.9% ( *n* = 66) of dentists indicated that the duration of discomfort lasted up to 3 weeks. 32.3% ( *n* = 61) of dentists reported that the pain lasted up to 8 weeks (4–8 weeks); 15.3% ( *n* = 29) of dentists indicated that the duration of pain lasted **>** 12 weeks. Other responses included 16.4% ( *n* = 31) who did not know how long DH lasted in their patients; one dentist could not recall and one dentist indicated that their patients experienced only occasional pain. There were two missing values.

Regarding their opinion on whether DH was a major impact on their patients’ QoL (Q.9), most dentists indicated that the condition did have a major impact on the QoL with 76.4% ( *n* = 146) of dentists stating “Yes,” 15.2% ( *n* = 29) stating “No” and 8.4% ( *n* = 16) stating they did not know.

When asked about the severity of DH on the QoL (Q.10), only 20.1% ( *n* = 33) of dentists indicated that the impact of DH was severe; most dentists (79.9%; *n* = 131) considered the impact to be no more than “mediocre” in nature. There were 27 (14.1%) missing values that were excluded from the analysis.

**Fig. 2:**
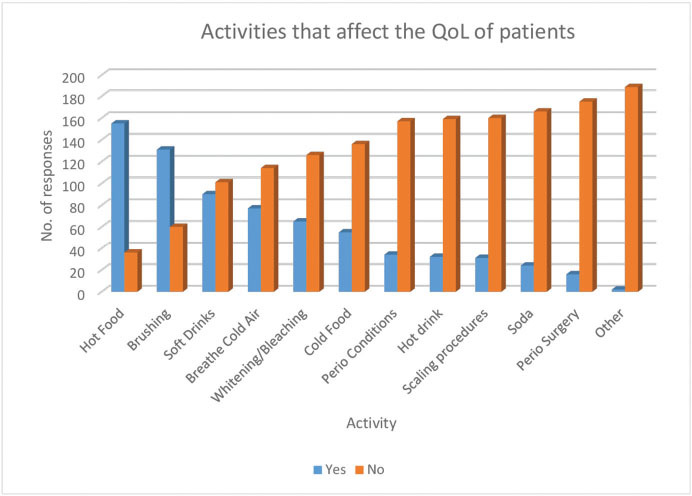
Activities that affect the quality of life (QoL) of patients (Q.11).

A follow-up question (Q.11) asked for information on the daily activities that may affect the QoL of patients with DH. These data are presented in Fig. 2 .

In response to Q.12 regarding whether their patients asked questions about DH, 42.9% ( *n* = 82) indicated they were asked “very often/often”; 56% ( *n* = 107) “sometimes/seldom” and 1% ( *n* = 2) “never.”

**Fig. 3:**
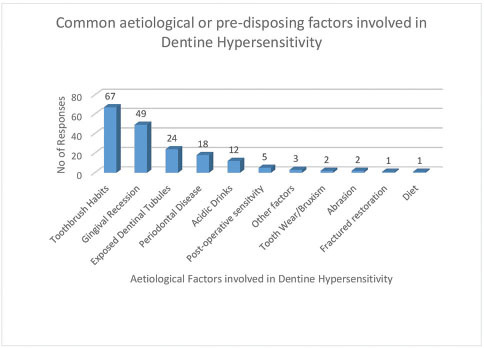
Factors involved in the etiology of dentine hypersensitivity (Q.13).

When asked to respond to Q.13, on the main factors in the etiology of DH, there were 184 responses from the participants and the main responses are presented in Fig. 3 .

When asked to respond to Q.14 on the underlying mechanism of DH, 57.4% ( *n* = 105) indicated that the “Hydrodynamic theory” was the underlying mechanism; 25.1% ( *n* = 46) identified the “odontoblast theory,” 9.3% ( *n* = 17) identified the “Gate theory.” Other responses included “other” (not specified) by 5.5% ( *n* = 10) of participants, a combination of mechanisms (hydrodynamic/odontoblast theory) by 2.2% ( *n* = 4) of participants, one participant (0.5%) was unsure. There were eight missing values (4.2%).

**Fig. 4:**
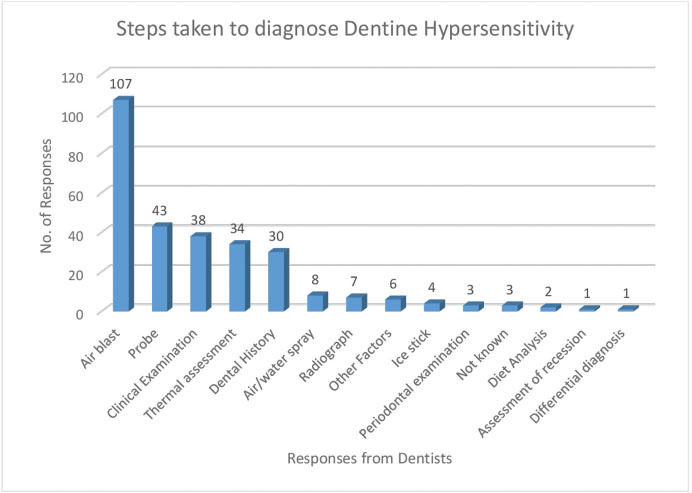
Steps taken by dentists to diagnose dentine hypersensitivity (Q.15).

In response to Q.15, the main responses to the diagnosis of DH are presented in Fig. 4 .

**Fig. 5:**
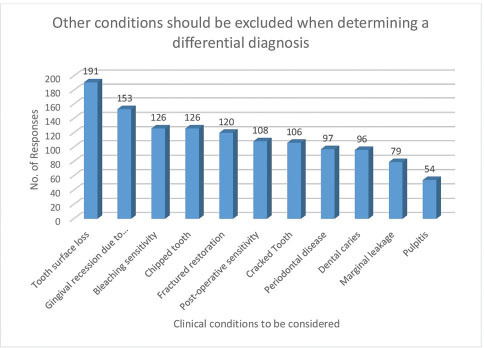
Clinical conditions to be excluded when determining a diagnosis of dentine hypersensitivity.

Fig. 5 presents the main responses regarding the dental conditions to be excluded when making a diagnosis (Q.16)

**Fig. 6:**
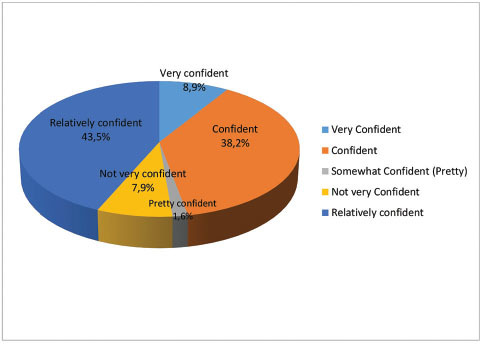
Dentists’ estimation of how confident they are of making a differential diagnosis of dentine hypersensitivity.

In response to Q.17 as to how confident they were in making a differential diagnosis, most dentists (92.1%; *n* = 176) claimed that they were confident in making a differential diagnosis of DH with 7.9% ( *n* = 15) stating they were not very confident ( Fig. 6 ).

**Fig. 7:**
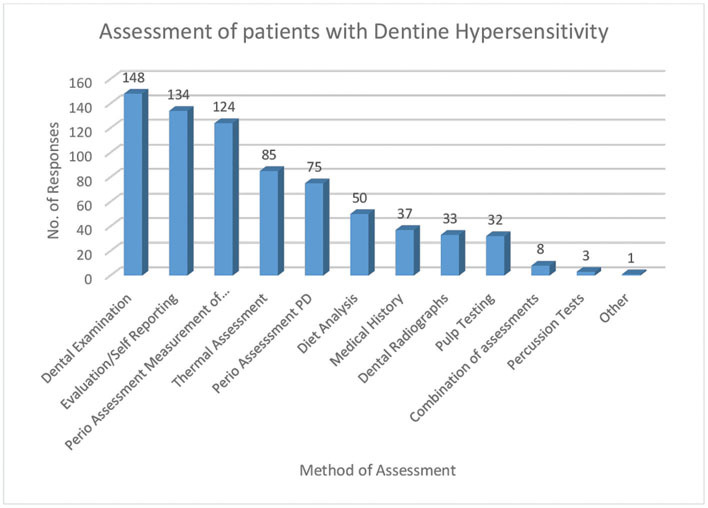
Dentists’ responses on how to clinically assess dentine hypersensitivity.

When asked to indicate how they would clinically assess or evaluate DH (Q.18), the dentists were given a range of options to choose from and the responses are presented in Fig. 7 .

**Fig. 8:**
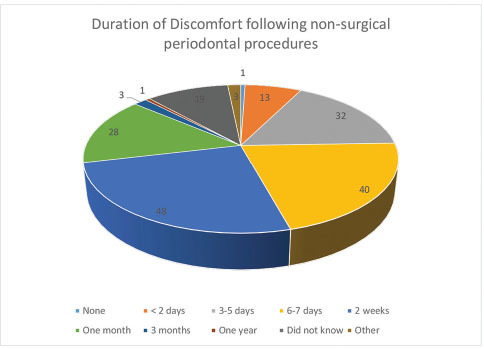
Duration of discomfort following nonsurgical periodontal procedures.

**Fig. 9:**
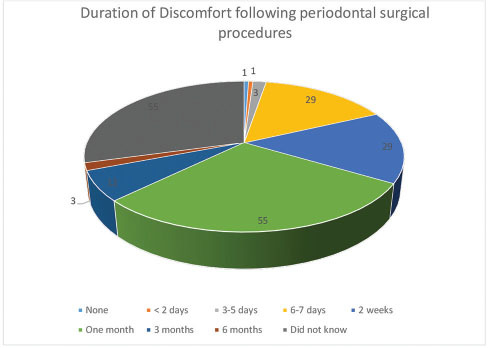
Duration of discomfort following periodontal surgical procedures.

In response to Q.19 on the duration of discomfort following nonsurgical periodontal procedures (scaling, debridement, and polishing), main responses are presented in Fig. 8 , while responses to Q.20 on the duration of discomfort following surgical periodontal procedures are depicted in Fig. 9 . When asked what advice they would provide to patients with DH (Q.21), the responses included (1) education on brushing (24%; *n* = 149), (2) at-home toothpastes or gels (23%; *n* = 143), (3) in surgery (professionally applied) options such as varnishes (16%; *n* = 100), (4) restorative dentistry (15%; *n* = 91), and a combination of options ( *n* = 3).

In response to Q.22 on whether they would recommend either in-office (professionally applied) or over-the-counter (at-home) (OTC) 59% ( *n* = 110) of the dentists indicated that they would recommend in-surgery (professionally applied) products such as a varnish to their patients. 86.6% ( *n* = 161) also indicated that they would recommend OTC products for home use. There were five missing values for these two categories. Of the recommendations for professional applied products or procedures ( *n* = 47), Duraphat varnish ( *n* = 29) was the most recommended product for DH. The other responses included air abrasion ( *n* = 2), Gel Kam ( *n* = 1), self-etching systems ( *n* = 2), bonding systems ( *n* = 4), and nonspecified treatment of gingival recession ( *n* = 9). When asked what OTC products they would recommend for home use, the following brands ( *n* = 54) were recommended: (1) Colgate Pro Relief ( *n* = 23), (2) Sensodyne ( *n* = 22), (3) Duraphat 5000 (4) and other nominated brands included Calmodent ( *n* = 2), Sensigel ( *n* = 2), and Soothe ( *n* = 1).

When asked whether they were confident in recommending home use products (OTC) (Q.23), most dentists indicated that they were confident or sure in recommending OTC products (83.6%; *n* = 158). 16.4% ( *n* = 31), however, indicated that they were not very confident in recommending OTC products for home use.

Responses to Q.24 relating to whether nondental problems may contribute to DH 60.2% ( *n* = 115) said that “non-dental problems” may contribute whereas 27.2% ( *n* = 52) did not think that these problems contributed to DH. Twelve percent ( *n* = 23) of dentists did not know whether these factors contributed to DH. There was one missing value. Only 20 responses were provided for this question with “Stress” ( *n* = 10) and “Psychological issues” ( *n* = 1) being the main nondental contributory factors. Other suggested factors included “Bruxism” ( *n* = 8) and “Malocclusion” ( *n* = 1).

When asked whether their patients frequently complied with professional advice when managing DH (Q.25), 79.4% ( *n* = 150) said their patients did comply with the advice provided, 4.2% ( *n* = 8) said that their patients did not comply with the advice, and 16.4% ( *n* = 31) did not know whether their patients complied with the advice. There were two missing values.

The last question (Q.26) asked whether dentists perceived a need for any patient leaflets/folders providing information on the problem and how to prevent it from becoming a problem or their patients. 88.3% ( *n* = 166) of dentists said there was a need for this type of literature with 11.7% ( *n* = 22) indicating that there was no need. There were three missing values and only four comments providing suggestions for the leaflets namely (1) factors associated with DH ( *n* = 2) and (2) information on how to treat DH ( *n* = 2).

## Discussion

Previous studies or reviews have indicated that dentists may be uncertain about the etiology, diagnosis, and effective management of dentine sensitivity/DH. 1 4 5 7 8 According to the 2003 Consensus Document on DH, 1 there are several concerns relating to the effective diagnosis and management of the condition such as (1) underestimating the prevalence of DH particularly for young adults, (2) screening of DH was not routinely conducted by dentists except when prompted by patients, (3) the failure to conduct a differential diagnosis to exclude other clinical conditions that have the same or similar features to that of DH, and (4) the apparent lack of confidence expressed by dentists in managing their patients’ pain. The questionnaire used in the present study was based on a previously validated questionnaire used in the United Kingdom relating to the understanding of DH, 8 14 15 which has been recently updated and used in several studies namely in Brazil, India, and Kuwait. 5 16 17

The response rate from the participants was 83% that in comparison to previous questionnaires of this nature was very good, 5 7 8 10 although it should be noted that the questionnaires were personally handed out by two of the investigators (CE/IB) to a selected group of dentists rather than using an online survey or mailing the questionnaire to the participants and this may have introduced some bias into the study. A comparable study of Australian private practice dentists by Amarasena et al 10 reported a lower response rate of 41.5%, although this study was based on a questionnaire mailed to randomly selected dentists on the Australian Dental Association membership list. One of the advantages of the questionnaire used in the present study was that it had been used and validated in several studies throughout the world. 5 8 14 15 16 17 The responses from the various questions in the present study appear to be consistent with the results of studies in other countries, although there are some differences with other published studies when the participants were asked about the etiological factors associated with DH as discussed below. 10 The authors also recognized that the questionnaire may be improved with a more prescriptive list of available products as used in other studies 10 18 rather than the use of open-ended questions to identify the products used or recommended by the dentist.

One of the problems in conducting questionnaire studies in to a participant's understanding or knowledge of a topic is that the reasons for a poor or low response rate may not be obvious. For example, one of the reasons could be due to a lack of knowledge and understanding by the participants and therefore a reluctance to return the questionnaire. 5 17 19 Alternatively, factors such as a lack of time to complete the questionnaire or simply considering that completing a questionnaire were not relevant to their practice needs or high on their list of priorities. 5

The prevalence of DH reported in the published literature varies depending on how the data was collected; according to Cunha-Cruz and Wataha 6 the best estimate of the prevalence is 10%. Several other investigators have also reported similar prevalence rates from dental practices 5 11 that is reasonably consistent with the data collected during the present study (39.8% of dentists indicated that one in 10 of their patients experienced discomfort from DH). The question of under-reporting of DH has also been raised by several investigators, 1 4 5 13 20 in particular as to whether the dentist or the patient initiated the conversation on DH; in the present study, 76.4% ( *n* = 146) of dentists indicated that their patients initiated the conversation on DH. Forty-four percent ( *n* = 84) of the dentists indicated that they initiated the conversation with 38.2% ( *n* = 73) of dentists who responded that they sometimes initiated the conversation about DH. According to Gillam, 4 DH does not appear to be considered by patients as a severe dental problem and as such do not appear to self-treat with home use (OTC) products or visit their dentist to resolve the problem. This would appear to suggest that the condition is fairly transient in nature with a limited impact on their QoL as indicated in the present study with 77.2% ( *n* = 127) of the dentists indicating that they considered discomfort from DH to last up to 8 weeks with only a “mediocre” impact on their patients’ QoL. One aberrant finding from the question on the activities that may impact on the patients’ QoL was that most dentists (68.6%; *n* = 131) considered incorrect tooth brushing to be an activity that would affect QoL. This finding is not consistent with several previous studies and most patients tend to have coping mechanisms when they experience unpleasant or painful sensations. 3 21 Another aberrant finding from the present study was that “hot” rather than “cold” products were considered to be a key trigger for DH that was at variance to most studies where “cold” was considered to be the predominant stimulus initiating DH. 10 19 21

Toothbrushing was also considered as an etiological or predisposing factor in DH that was a similar finding in several studies. 5 15 Abrasion and gingival recession were also considered by dentists in the present study and to a lesser extent erosion despite being considered as a primary cause of DH. 1 (DH/T), erosion 28.9% (dentists), and 26% (DHT) were considered as alternative causes. Over half of the dentists indicated that the “Hydrodynamic theory” was the underlying mechanism of DH that is reasonably consistent with other studies. 5 10 14 15 16

When asked to identify how they would identify and examine DH in the clinical environment, dentists indicated that they would mainly rely on “clinical examination” and “self-reporting of DH from patients and use” either the “air blast” or “probing” methods that are consistent with the results from other studies. 5 10 14 15 16

One of the concerns from the consensus conference document 1 was that there were several key knowledge gaps in the responses from the participants in the survey. For example, the failure to consider a differential diagnosis, even though DH is by definition a diagnosis of exclusion and the apparent lack of confidence in managing their patients’ pain. In the present study, the dentists claimed that they were confident in conducting a differential diagnosis as well as having confidence in recommending both OTC and professionally applied products. Of the other dental conditions to consider when making a diagnosis of DH, the main responses were “Tooth surface loss,” “Gingival recession” as a result of “periodontal disease and/or its treatment,” and “Bleaching sensitivity” that was at variance with other studies where dental caries were also considered as a dental condition to be considered when making a diagnosis, 5 10 although reasonably similar to the study by Kopycka-Kedzierawski et al. 13

It is also accepted that patients may experience postoperative sensitivity from dental procedures such as nonsurgical and surgical procedures and that this discomfort may resolve within 1 to 2 weeks for nonsurgical procedures and up to 8 weeks following surgical procedures. 22 The results from the present study are reasonably consistent with the published figures.

83.6% of dentists indicated that they were confident in recommending OTC products for home use and they indicated that they would recommend OTC products such as “Colgate Pro Relief” and “Sensodyne” with a similar number of dentists recommending a professionally applied product such as a varnish (“Duraphat”) for their patients. Compared with other studies, the responses from the present study were not as detailed and this may have been due to open-ended rather than a closed question approach to this question, 10 18 although the conclusions about the recommendations for treatment were similar in these studies.

The present study highlighted several differences from previous studies and it was apparent there is a need for additional education strategies to be practiced in everyday dental practice in particular a greater focus on the importance of the prevention in the management of DH as well as in the diagnosis and management of the condition. Furthermore, the dentist should recognize that there are other forms of chronic pain when considering a differential diagnosis as well as the need to have a monitoring strategy when managing DH. 1 20 In relation to initiating a management strategy, it is worth considering implementing a training program to enable dentists to provide a standardized reporting as used in the USA PEARL and National Practice Based-Research Network (PBRN) programs. 5 11 13 18

## Conclusion

The results of the present study would appear to suggest that in terms of knowledge and understanding of DH, there is still confusion concerning some aspects of the diagnosis and management of the condition. There is clearly a need for additional education strategies to be practiced in everyday dental practice, in particular a greater focus on the importance of prevention in the management of DH as well as in the diagnosis and management of the condition.

## References

[1] Canadian Advisory Board on Dentin Hypersensitivity Consensus-based recommendations for the diagnosis and management of dentin hypersensitivity J Can Dent Assoc 2003 69 221 226 12662460

[2] Bekes K John M T Schaller H G Hirsch C Oral health-related quality of life in patients seeking care for dentin hypersensitivity J Oral Rehabil 2009 36 01 45 51 19207369 10.1111/j.1365-2842.2008.01901.x

[3] Gibson B Boiko O V Baker S R The everyday impact of dentine sensitivity: personal and functional aspects Soc Sci Dent 2010 1 11 21

[4] Gillam D G Current diagnosis of dentin hypersensitivity in the dental office: an overview Clin Oral Investig 2013 17 01 S21 S29 10.1007/s00784-012-0911-1 PMC3586159 23296425

[5] Pereira R Gillam D G Bapatla S Satyamurthy P Awareness of dentine hypersensitivity among general dental practitioners in Mumbai, India J Odontol 2018 2 103

[6] Cunha-Cruz J Wataha J C The burden of dentine hypersensitivity Oxford Elsevier 2014 34 44

[7] Schuurs A H Wesselink P R Eijkman M A Duivenvoorden H J Dentists’ views on cervical hypersensitivity and their knowledge of its treatment Endod Dent Traumatol 1995 11 05 240 244 8625939 10.1111/j.1600-9657.1995.tb00496.x

[8] Gillam D G Bulman J S Eijkman M A Newman H N Dentists’ perceptions of dentine hypersensitivity and knowledge of its treatment J Oral Rehabil 2002 29 03 219 225 11896837 10.1046/j.1365-2842.2002.00812.x

[9] Orchardson R Gillam D G Managing dentin hypersensitivity J Am Dent Assoc 2006 137 07 990 998, quiz1028–1029 16803826 10.14219/jada.archive.2006.0321

[10] Amarasena N Spencer J Ou Y Brennan D Dentine hypersensitivity - Australian dentists’ perspective Aust Dent J 2010 55 02 181 187 20604761 10.1111/j.1834-7819.2010.01223.x

[11] Northwest Practice-based Research Collaborative in Evidence-based DENTistry Cunha-Cruz J Wataha J C Heaton L J The prevalence of dentin hypersensitivity in general dental practices in the northwest United States J Am Dent Assoc 2013 144 03 288 296 23449905 10.14219/jada.archive.2013.0116 PMC3819160

[12] Benoist F L Ndiaye F G Faye B Bane K Ngom P I Ndong P M Knowledge of and management attitude regarding dentin hypersensitivity among dentists from a West African country J Contemp Dent Pract 2014 15 01 86 91 24939271 10.5005/jp-journals-10024-1493

[13] National Dental PBRN Collaborative Group Kopycka-Kedzierawski D T Meyerowitz C Litaker M S Management of dentin hypersensitivity by National Dental Practice-Based Research Network practitioners: results from a questionnaire administered prior to initiation of a clinical study on this topic BMC Oral Health 2017 17 01 41 28086862 10.1186/s12903-017-0334-0 PMC5237301

[14] Hatton J Kumar K Gillam D G Knowledge of dental undergraduates and dentists in treating dentine hypersensitivity. J Dent Res 2012. doi: 10.5005/jp-journals-10024-1493

[15] Francisconi L Calabria M Knowledge of Brazilian Dentists and Students in Treating Dentine Hypersensitivity J Dent Res [Spec Issue A] 2013 92: Abstract no. 2193

[16] Dashti N AAA A survey of the professional opinions of Kuwaiti dentists for the treatment and management of dentine hypersensitivity: questionnaire based study. D Clin Dent Research report QMUL London 2018

[17] Blanchard P Wong Y Matthews A G Restoration variables and postoperative hypersensitivity in Class I restorations: PEARL Network findings. Part 2 Compend Contin Educ Dent 2013 34 04 e62 e68 23627487 PMC4392922

[18] Gillam D G Orchardson R Advances in the treatment of root dentine sensitivity: mechanisms and treatment principles Endod Topics 2006 13 13 33

[19] Gillam D G A new perspective on dentine hypersensitivity – guidelines for general dental practice Dent Update 2017 44 01 33 36,39–42 29172308 10.12968/denu.2017.44.1.33

[20] Gillam D G Seo H S Bulman J S Newman H N Perceptions of dentine hypersensitivity in a general practice population J Oral Rehabil 1999 26 09 710 714 10520145 10.1046/j.1365-2842.1999.00436.x

[21] Lin Y H Gillam D G The prevalence of root sensitivity following periodontal therapy: a systematic review Int J Dent 2012 2012 407023 23193405 10.1155/2012/407023 PMC3501835

[22] Cunha-Cruz J Wataha J C Zhou L Treating dentin hypersensitivity: therapeutic choices made by dentists of the northwest PRECEDENT network J Am Dent Assoc 2010 141 09 1097 1105 20807910 10.14219/jada.archive.2010.0340 PMC3052855

